# Molecular Pathways Bridging Frontotemporal Lobar Degeneration and Psychiatric Disorders

**DOI:** 10.3389/fnagi.2016.00010

**Published:** 2016-02-01

**Authors:** Roberta Zanardini, Miriam Ciani, Luisa Benussi, Roberta Ghidoni

**Affiliations:** Molecular Markers Laboratory, IRCCS Istituto Centro San Giovanni di Dio, FatebenefratelliBrescia, Italy

**Keywords:** BDNF, progranulin, biomarkers, frontotemporal dementia, psychiatric disorders, neurodegenerative diseases

## Abstract

The overlap of symptoms between neurodegenerative and psychiatric diseases has been reported. Neuropsychiatric alterations are commonly observed in dementia, especially in the behavioral variant of frontotemporal dementia (bvFTD), which is the most common clinical FTD subtype. At the same time, psychiatric disorders, like schizophrenia (SCZ), can display symptoms of dementia, including features of frontal dysfunction with relative sparing of memory. In the present review, we discuss common molecular features in these pathologies with a special focus on FTD. Molecules like Brain Derived Neurotrophic Factor (BDNF) and progranulin are linked to the pathophysiology of both neurodegenerative and psychiatric diseases. In these brain-associated illnesses, the presence of disease-associated variants in *BDNF* and progranulin (*GRN*) genes cause a reduction of circulating proteins levels, through alterations in proteins expression or secretion. For these reasons, we believe that prevention and therapy of psychiatric and neurological disorders could be achieved enhancing both BDNF and progranulin levels thanks to drug discovery efforts.

## Introduction

Over the past 10 years frontotemporal lobar degeneration (FTLD) has emerged, along with Alzheimer disease (AD), as a leading cause of dementia developing in midlife or earlier (Ratnavalli et al., 2002; Onyike and Diehl-Schmid, 2013). Identification of FTLD-causing genetic alterations has been complex due to the variety of genes involved and the different protein functions. While the majority of FTLD cases are sporadic, approximately 40% of patients have a positive family history (Gass et al., 2012) and about 10% show a clear autosomal-dominant inheritance, suggesting the involvement of genetic components in the etiology of this disorder (Snowden et al., 2002; Seelaar et al., 2008; Rohrer et al., 2009; Benussi et al., 2015). Genetic studies have identified several genes linked to FTLD: microtubule-associated protein tau (*MAPT*), progranulin (*GRN*), TAR DNA-binding protein 43 (*TDP-43*), valosin-containing protein (*VCP*), charged multivesicular body protein 2B (*CHMP2B*), fused in sarcoma (*FUS*), and the hexanucleotide repeat expansion in intron 1 of the chromosome 9 open reading frame 72 (*C9ORF72*; Hutton et al., 1998; Watts et al., 2004; Skibinski et al., 2005; Baker et al., 2006; Cruts et al., 2006; Broustal et al., 2010; DeJesus-Hernandez et al., 2011; Renton et al., 2011; Rohrer et al., 2015). The neuropathology of FTLD is heterogeneous and subtypes can be distinguished depending on the protein deposited in inclusion bodies in the central nervous system (CNS; Mackenzie et al., 2010). These protein inclusions within neurons and glial cells together with degeneration of the brain’s frontal and temporal lobes are considered the primary neuropathological hallmark of FTLD (Gass et al., 2012). The most common clinical subtype of frontotemporal dementia is the behavioral variant (bvFTD), which is characterized by progressive behavioral changes, such as disinhibition, apathy/inertia, loss of sympathy/empathy, perseverative, stereotyped and compulsive/ritualistic behavior and hyperorality (Rascovsky et al., 2011). These psychiatric symptoms in FTLD mutation carriers can be very similar to those seen in non-neurodegenerative psychiatric illness (Block et al., 2015). Thus, bvFTD can mimic several psychiatric conditions, such as mood disorders (MD), obsessive–compulsive disorder (OCD) and schizophrenia (SCZ; Wylie et al., 2013). In parallel, some psychiatric disorders, notably SCZ, can progress to a syndrome of dementia that includes features of frontal dysfunction with relative sparing of memory (Friedman et al., 2001; Harvey, 2012). Moreover, bvFTD behavioral symptoms are often controlled by therapies that are available for psychiatric disorders, while no medication has been found to stabilize or improve their cognitive dysfunction (Vossel and Miller, 2008). A similar overlap of symptoms with psychiatric disorders has been observed not only in bvFTD, but also in other forms of dementia such as AD and Lewy Bodies Dementia (LBD). Neuropsychiatric symptoms, such as apathy, depression, delusion, aggression, physical and verbal agitation, are frequent in LBD and AD, even in the early stages of the disease, and are very distressing to patient caregivers (Nagahama et al., 2010; Lyketsos et al., 2011).

There is a pressing need to identify molecular pathways underlying neuropsychiatric symptoms in psychiatric disorders and dementias. Molecular links between neurodegeneration and psychiatric phenotype have been reported: (i) a recent study shows a decrease of progranulin plasma levels in subjects with bipolar disorder (BD; Kittel-Schneider et al., 2014); (ii) new data have suggested a role of amyloid-beta (Aβ) oligomers in memory impairment, depressive and anhedonic behavior in mice (Ledo et al., 2013); and (iii) transgenic mice overexpressing alpha-synuclein—involved in Parkinson’s disease and LBD—show late-onset anxiety behavior (Campos et al., 2013) and extracellular alpha-synuclein oligomers modulate synaptic transmission and impair Long Term Potentiation (LTP) via NMDA-receptor activation (Diógenes et al., 2012). The serotonergic system, classically involved in the depression/anxiety mechanisms, is now proposed to regulate Aβ precursor protein (APP) activity and metabolism (Shen et al., 2011), while Brain Derived Neurotrophic Factor (BDNF) has a relevance not only in MD, but also in depression-associated cognitive decline (Diniz et al., 2014). *In vivo* evidence suggests that Aβ aggregation dramatically influence BDNF cerebrospinal fluid (CSF) level in transgenic AD mouse models (Peng et al., 2009), underlining the importance of this neurotrophic factor in AD pathogenesis, as already suggested by genetic studies (Ventriglia et al., 2002; Chen et al., 2014). Moreover, a peculiar CSF Aβ peptide signature was associated to cognitive decline in SCZ, suggesting a dysmetabolism of APP also in this psychiatric disorder (Frisoni et al., 2011; Albertini et al., 2012). In addition, experimental studies showed that the levels of progranulin were significantly correlated with amyloid amount in mouse models of AD, detecting an increase of its levels in mice with extensive dense-core plaques (Minami et al., 2014).

Thus, mechanisms underlying neurodegenerative diseases and psychiatric disorders converge on the same proteins within different pathways. In this review, we will focus on the role of BDNF and progranulin in these pathologies.

## Our Protagonists

BDNF is the most studied and characterized neurotrophin in the CNS and it has received remarkable attention from clinicians because of its importance in the development and maintenance of normal brain functions. In humans, the *BDNF* gene is located on the short arm of chromosome 11p13 (Pruunsild et al., 2007). The gene comprises 11 exons, each coding for the 5′ untranslated region, spliced to a common downstream 3′ exon IX. BDNF is the result of translation of at least 34 mRNA transcripts and its expression is under the control of nine alternative tissue-specific promoters (Pruunsild et al., 2007). BDNF peptide is synthesized as its pro-isoform (pro-BDNF); proteolytical cleavage into its mature form occurs within the neuron or extracellularly, after secretion (Barker, 2009). Whereas pro-isoforms such as pro-BDNF bind to p75 neurotrophin receptor (p75^NTR^), the mature neurotrophins bind to protein-kinase neurotrophin receptors—namely tropomyosine-related kinase (Trk) receptors (Huang and Reichardt, 2003). BDNF binds to Trk B of the family of Trk receptors, a process that activates survival mechanisms in the CNS such as proliferation, growth, and neuroplasticity (Islam et al., 2009; Nguyen et al., 2009). In contrast, pro-BDNF activates apoptotic pathways after binding to p75^NTR^ (Teng et al., 2005) and induce neuronal remodeling including axonal and dendritic pruning (Barker, 2009; Koshimizu et al., 2009). BDNF, also thanks to its notable activity-dependent regulation of expression and secretion, seems to play an important role in neuronal plasticity, defined as a set of mechanisms that may conduct to neuronal reshaping, birth of new neurons and formation of novel synapses (Bramham and Messaoudi, 2005). This is especially important since growing evidence suggests a role for BDNF in the pathophysiology of brain-associated illnesses including both neurodegenerative and psychiatric diseases (Nagahara and Tuszynski, 2011).

Progranulin is a multi-functional secreted adipokine of 593 amino acids with trophic, anti-inflammatory and growth factor-like properties, characterized by dynamic changes in the expression level, glycosylation status and cellular localization during development and in the adult organism (Daniel et al., 2000, 2003; Petkau et al., 2010). The human progranulin gene (*GRN*) is located on chromosome 17q21.32 and it is composed of 12 protein-coding exons. Alternative splicing and exon skipping define three different progranulin isoforms (Bhandari and Bateman, 1992; Bhandari et al., 1992). The protein is characterized by a signal sequence and a series of seven and a half granulin repeats (De Muynck and Van Damme, 2011) separated by interlinked spacer regions, which are composed of highly conserved tandem repeats of a unique 12-cysteine motif (Shoyab et al., 1990; Bhandari and Bateman, 1992; Bhandari et al., 1992). After secretion, in peripheral tissues the proteolytic cleavage of progranulin at the intra-linker spacer regions give rise to several ~6 kDa peptides called granulins (Butler et al., 2008; Kessenbrock et al., 2008; Bai et al., 2009). However, the full length progranulin can be preserved from cleavage by secretory leukocyte protease inhibitor (SLPI; Zhu et al., 2002) and high-density lipoprotein (HDL)/Apolipoprotein A-I complex (Okura et al., 2010). Both progranulin precursor and its cleavage fragments are biologically active in a series of physiologic and pathological processes, showing opposing roles in different biological functions (De Muynck and Van Damme, 2011). Progranulin protein has many roles inside and outside the brain, acting through the extracellular signal-regulated kinase and phosphatidylinositol-3-kinases pathways (Toh et al., 2011). Progranulin is involved in multiple biological functions, such as in survival, migration and regulation of cellular proliferation, wound repair and inflammation, and it has also been speculated to play a role in excitotoxicity and synaptic transmission (Guo et al., 2010; Tapia et al., 2011). Specifically, in the CNS, progranulin seems to play a significant role in maintaining physiological functions because it has been observed that it has neuroprotective effects (Van Kampen et al., 2014) and neurotrophic activity *in vitro* and *in vivo* model systems (Van Damme et al., 2008; Gao et al., 2010; Laird et al., 2010). Multiple evidence supports that progranulin is involved in different pathological conditions, such as neurodegenerative, psychiatric and metabolic disorders, thus becoming a fascinating target for therapy (Philips et al., 2010; Petkau and Leavitt, 2014; Kanazawa et al., 2015).

Taken together these data highlight neurotrophic properties for both BDNF and progranulin with a significant role in synaptic density and morphology. Further cellular and molecular studies indicate similar functions and participation in common pathways for our protagonists. The identification of sortilin 1 (SORT1) as the principal neuronal receptor for progranulin was important for our understanding of progranulin biology. SORT1 is a sorting protein involved in endocytosis and transport to trans-golgi network and endosomes: when progranulin is bound to SORT1 it is immediately endocytosed and delivered to the lysosome. Indeed, the uptake of progranulin via SORT1 defines a substantial reduction of extracellular progranulin levels (Petersen et al., 1997). Recently, a determinant role for SORT1 has been described in the pathogenesis of FTLD: deletion of this receptor results in a 2.5 to 5-fold increase in progranulin levels and reversal of progranulin deficiency in the *GRN* ± FTLD model (Hu et al., 2010). Thus, SORT1 is an important regulator of progranulin extracellular levels, while the neurotrophic and neuroprotective effects of progranulin are independent from its uptake mediated by SORT1 receptor (De Muynck et al., 2013). Of note, SORT1 also plays an important role in the biological function of BDNF. It plays an essential role in the regulation of BDNF levels through modulation of both anterograde and lysosomal trafficking (Chen et al., 2005; Evans et al., 2011). Moreover, SORT1 can form a complex with p75^NTR^ and ProBDNF to induce cell death; on the other hand, SORT1 has also been implicated in anterograde trafficking of the Trk receptors from the soma to the nerve terminal, thereby positively regulating BDNF signaling and cell survival (Vaegter et al., 2011).

The presence of common function of the two proteins is further supported by a recent study reporting that progranulin is co-localized with dense-core vesicle markers and is further recruited to synapses following neuronal activity. Furthermore, the authors observed that, similarly to what is described for BDNF, progranulin is secreted from axons and dendrites in an activity-dependent manner with different temporal profiles of secretion. Treatment of neurons with recombinant progranulin was shown to increase synapse density, while decreasing the size of the presynaptic compartment and specifically the number of synaptic vesicles per synapse (Petoukhov et al., 2013). In parallel, a study has demonstrated that acute and gradual increases in BDNF concentration activate different intracellular cascades, leading to differences in spine morphology (Ji et al., 2010). These results suggest that it is not only the concentration of secreted BDNF that it is important for the regulation of neuronal function and morphology, but also the manner in which it is secreted: constitutively or in response to neuronal activity. It is possible that constitutive and regulated secretion of progranulin also differentially regulates synapse structure and function.

## BDNF and Progranulin in FTLD and Related Neurodegenerative Diseases

Current hypotheses state that the crucial point in the progression of neurodegenerative diseases is driven by deterioration of synapses. This is supported by synapse loss being observed as a major pathophysiological hallmark in all neurodegenerative diseases. BDNF, thanks to its role in synaptogenesis, becomes the best pawn on the chessboard with respect to counteracting synaptic function loss. A reduction of hippocampal BDNF levels not only reduced the number of synapses, but also impaired LTP and caused deficits in the formation and consolidation of hippocampus-dependent memory (Linnarsson et al., 1997; Ma et al., 1998; Mu et al., 1999).

Neurotrophins can protect synapses against various toxic insults in animal models of neurodegenerative diseases, such as AD, Huntington’s disease, Amyotrophic Lateral Sclerosis and Parkinson’s disease (PD; Nagahara and Tuszynski, 2011). Remarkably, BDNF has been shown to protect and/or repair hippocampal neurons and synapses despite Aβ accumulation and neuronal toxicity in a mouse model of AD (Nagahara et al., 2009) and to rescue plasticity deficits induced by synthetic Aβ oligomers in rat hippocampal slices *ex vivo* (Zeng et al., 2010). This suggests a therapeutic potential in presence of pathogenic factors (Lu et al., 2013). Furthermore, transplantation of neural stem cells (NSC) secreting BDNF in AD model mice also improved cognitive function (Blurton-Jones et al., 2009). BDNF-based therapy is therefore increasingly expected to ameliorate the symptoms of AD (Nagahara et al., 2009; Peng et al., 2009; Devi and Ohno, 2012; Adachi et al., 2014). Decreased expression levels of BDNF protein and mRNA have been consistently reported in the hippocampus and cortex of individuals with AD (Phillips et al., 1991; Connor et al., 1997; Hock et al., 2000; Holsinger et al., 2000) as well as in serum (Laske et al., 2007). Belrose et al. (2014) analyzed neurotrophins alterations in patients with different tauopathies, observing a significant decrease in BDNF mRNA and protein levels in Pick’s disease and corticobasal degeneration. A further study on the role of this neurotrophin in different neurological diseases observed lower levels of BDNF protein in serum of FTLD AD, LBD and vascular dementia patients, whereas the protein was increased in PD patients (Ventriglia et al., 2013). In the *BDNF* gene, a functional polymorphism (SNP, rs6265) has been characterized, producing a valine (Val) to methionine (Met) substitution at the codon 66 (Val66Met) in the proBDNF region. The valine-for-methionine substitution is associated with inefficiency inactivity-dependent transportation of BDNF mRNA (Chiaruttini et al., 2009) and protein to dendrites (Egan et al., 2003; Chen et al., 2004), thus contributing to reductions in dendritic density, deficits in synaptic connectivity, and neurocognitive deficits (Egan et al., 2003; Chen et al., 2004). Indeed, inheritance of the BDNF Met allele might be expected to confer susceptibility to or modifying the expression of conditions that impact adversely on hippocampal function, as measured also by functional magnetic resonance imaging (Egan et al., 2003; Hariri et al., 2003; Dempster et al., 2005; Nagahara and Tuszynski, 2011).

It has been reported that BDNF Val66Met polymorphism may play a role as a genetic risk factor and diseases modulator for FTLD and may drive a selective damage in specific brain region affected by the disease (Borroni et al., 2012). Due to its role on neuronal function and survival in different systems in the CNS, BDNF is an interesting candidate for drug discovery. Strategies that aim to achieve safe and effective, dose-continuous delivery of BDNF in the brain, as drug-induced increasing BDNF, exercise and diet, epigenetic or peptide mimetic of BDNF, seem promising (Nagahara and Tuszynski, 2011).

Loss-of-function mutations in *GRN* are a common cause of FTLD. Since 2006, more than 70 distinct pathogenic mutations have been identified in the *GRN* gene, accounting for up to 20% of familial and 5% of sporadic FTLD cases (Cruts et al., 2012). Most of these pathogenic alterations are characterized as nonsense, frameshift and splice site mutations causing a premature stop codon, even if other mechanisms have also been observed (Ghidoni et al., 2008; Gass et al., 2012). All pathogenic mutations identified cause disease by protein haploinsufficiency (Baker et al., 2006; Ghidoni et al., 2008; Finch et al., 2009; Sleegers et al., 2009; Cruts et al., 2012) leading to a ~50% or greater decrease in progranulin levels in the blood, unaffected brain regions and CSF of *GRN* mutated subjects (Coppola et al., 2008; Ghidoni et al., 2008, 2012; Finch et al., 2009; Sleegers et al., 2009). Furthermore, circulating progranulin levels have been proposed as a useful biomarker for a quick and inexpensive large-scale screening of *GRN* mutation carriers (Ghidoni et al., 2012). Though all *GRN* null mutations cause the disease through this common mechanism, linked to the degradation of RNA through non-sense mediated decay (Baker et al., 2006; Cruts et al., 2006), the spectrum of clinical presentations associated with mutations in *GRN* is heterogeneous: however, the bvFTD is the most common clinical presentation, characterized by progressive behavioral changes (Gass et al., 2006; Rademakers et al., 2007; Beck et al., 2008; Le Ber et al., 2008; Benussi et al., 2009).

In addition to null mutations, *GRN* polymorphic variants, located in the promoter and in the 3′ untranslated region, have been described to increase the risk to develop FTLD, possibly by influencing the transcription of the gene (Rademakers et al., 2008; Galimberti et al., 2010). The *GRN* gene expression is also regulated by DNA methylation in its core promoter region (Banzhaf-Strathmann et al., 2013). Interestingly, an increased methylation of *GRN* promoter region was described in cells and brains from FTLD, specifically in patients with bvFTD clinical variant (Banzhaf-Strathmann et al., 2013; Galimberti et al., 2013). Different lines of *GRN*-knockout mice have been produced showing phenotypes that qualifiy them as useful models of FTD (Kayasuga et al., 2007; Yin et al., 2010; Ghoshal et al., 2012). Specifically, Yin et al. (2010) obtained progranulin-deficient mice characterized by phenotypic alterations that resembles what is typically observed in patients with bvFTD. All these considerations suggest that progranulin represents a promising target for treatment of different neurodegenerative diseases (Boxer et al., 2013; Van Kampen et al., 2014).

Potential treatments to counteract progranulin loss in neurodegeneration are in development because they could represent a promising therapeutic strategy to decelerate neurodegenerative progression or at least to alleviate the symptoms associated with disease (Gass et al., 2012; Jing et al., 2015). It has been observed that increasing progranulin levels might reduce neuronal vulnerability to injury, inflammation and other insults associated to increased risk of cell death and diseases such as FTLD (Gass et al., 2012). Several chemical compounds, such as Bafilomycin A1 (BafA1), alkalizing compounds and suberoylanilide hydroxamic acid (Capell et al., 2011; Cenik et al., 2011), and genetic factors, including different microRNAs and also different genetic variants (Cruchaga et al., 2011; Finch et al., 2011; van der Zee and Van Broeckhoven, 2011), have recently been identified as enhancer of *GRN* expression, demonstrating therapeutic potential in progranulin associated- FTLD. Maybe the way to develop an efficacious therapy is still far away, but these first results are encouraging.

## BDNF and Progranulin in Psychiatric Disorders

Growing evidences suggest a role for BDNF in the pathophysiology of brain-associated illnesses such as OCD (Maina et al., 2010), attention deficit-hyperactivity disorder (Scassellati et al., 2014), eating disorders (Monteleone et al., 2006) substance abuse (Zanardini et al., 2011) as well as MD, BD and SCZ (Sen et al., 2008; Adachi et al., 2014; Polyakova et al., 2015). Rodent models of depression show reduced expression levels of BDNF and its receptor Tropomyosin receptor kinase B (TrkB). Different types of stressors on rodents can reduce expression levels of BDNF in amygdala, cortex and hippocampus (Stepanichev et al., 2014). Of note, direct BDNF infusion into the rat midbrain induced antidepressant-like effects (Siuciak et al., 1997). Patients suffering from depression exhibit specific BDNF levels reduction in hippocampus and serum (Dwivedi et al., 2003; Bocchio-Chiavetto et al., 2010). Treatments with antidepressant drugs reversed this condition (Sen et al., 2008); this effect was also observed after repetitive transcranial magnetic stimulation, a non-pharmacological intervention (Zanardini et al., 2006). The role of BDNF in BD is unclear, probably because the disease is characterized by different mood states in which the expressiuon of neurotrophin is modulated (Cunha et al., 2006; Yoshimura et al., 2006; de Oliveira et al., 2009; Tramontina et al., 2009). In fact, a recent systematic review and meta-regression analysis reported that peripheral BDNF could be used as a biomarker for mood states and disease progression in BD (Fernandes et al., 2011). Mood stabilizers such as lithium or valproic acid are utilized to treat BD patients and as happens also for antidepressant, increase the BDNF expression (Hashimoto et al., 2002; Chen and Manji, 2006; Sanacora, 2008). Furthermore, BDNF is also decreased in the prefrontal cortex and hippocampus in an animal model of mania (Frey et al., 2006). In addition, The V66M BDNF allele is strongly associated with BD (Neves-Pereira et al., 2002; Sklar et al., 2002; Lohoff et al., 2005). Moreover, Cattaneo et al. (2010) describe an interesting reduction of BDNF in amniotic fluid of Met carriers (Val/Met and Met/Met) as compared to non carriers (Val/Val), supporting the involvement of this polymorphism in behavioral and functional brain individual differences. Animal studies show that BDNF controls the development and activity of neurotransmitter systems implicated in psychotic disorders (Tanaka et al., 1997; Mattson, 2008). Indeed, neurodevelopmental models of SCZ suggest that reduced BDNF may affect synaptic efficiency and connectivity in SCZ that is believed to underlie core behavioral signs and symptoms of the disease (Lipska et al., 2003; Toro et al., 2007; van Haren et al., 2008). There is no widespread agreement on the degree of peripheral BDNF reduction in SCZ, as measured in blood serum or plasma. Most studies report reduced BDNF levels measured in blood from SCZ patients (Toyooka et al., 2002; Pirildar et al., 2004; Grillo et al., 2007; Mackin et al., 2007; Ikeda et al., 2008; Lee and Kim, 2009; Xiu et al., 2009; Jindal et al., 2010), whereas two studies describe higher levels of BDNF concentration in serum of schizophrenic patients compared with healthy controls (Gama et al., 2007; Reis et al., 2008). There are also studies that report no significant difference in BDNF plasma levels in non-medicated SCZ patients compared with controls, while increased BDNF levels observed in these same subjects following antipsychotic treatment (Lee and Kim, 2009). Some studies evidence altered BDNF mRNA and protein levels in prefrontal cortical regions of post-mortem brain tissue of schizophrenic subjects (Durany et al., 2001; Mellios et al., 2009; Wong et al., 2010), with variation reported across different brain regions (Durany et al., 2001; Hashimoto et al., 2005). Several researchers have focused their attention on DNA methylation as a repressive mechanism for *BDNF* gene expression, potentially linked to pathogenesis of mental diseases (Tsankova et al., 2007; Mill et al., 2008; Fuchikami et al., 2011). Reduced *BDNF* gene expression in combination with a considerable increase of promoter methylation levels, was selectively detected in BD type II subjects (BD-II), but not in BD I group (BD-I) with respect to controls. This result confirms a prior study demonstrating that BDNF expression is decreased in chronic or later stages of BD compared with early stages of disease (Kauer-Sant’Anna et al., 2009). This suggests that attempting to therapies that may be useful to restore a correct degree of methylation could be beneficial (Nan et al., 1998; Fuks et al., 2000; Garcia-Manero et al., 2006; Candelaria et al., 2007). It has been largely demonstrated that mutations in *GRN* cause FTLD. It is less known that common genetic polymorphisms in this gene are associated with SCZ and bipolar affective disorder (Rademakers et al., 2008; Momeni et al., 2010; Schoder et al., 2010; Galimberti et al., 2012, 2014). A systematic literature analysis reveals that psychosis is a frequent symptom in FTD in the presence of genetic mutations in *GRN* and C9ORF72 genes (Shinagawa et al., 2014); in *C9ORF72* carriers, psychotic symptoms can even predominate at onset and lead to a diagnosis of OBC, SCZ or BD (Rohrer et al., 2015). At present, the effects of progranulin impairment in psychiatric diseases have not yet been studied in specific animal models. However, since (i) mutations in *GRN* cause bvFTD and (ii) *GRN* knock-out models are characterized by prominent behavioral disturbances, the study of such animal models could be useful to understand the molecular mechanisms underlying psychiatric disorders (Roberson, 2012). Galimberti et al. performed an association study in which they analyzed the correlation between progranulin gene variations and the risk for developing BD in patients with BD-I and BD-II (Galimberti et al., 2012, 2014): the specific *GRN* SNP rs5848, which is associated with variation in progranulin levels (Galimberti et al., 2014), resulted to be a protective factor associated with BD-I, but not with BD-II, probably due to different pathogenic mechanisms among the two subtypes of diseases. In addition, they observed progranulin plasma levels significantly lower in the whole BD population compared with controls, even if they did not exclude the effect of the therapy on progranulin levels. This previous finding was confirmed by a larger replication analysis (Kittel-Schneider et al., 2014). Moreover, also in this BD cohort it has been observed that age has a significant effect on progranulin levels, with increasing levels in higher age, data which is coherent with what previously reported in FTLD patients (Ghidoni et al., 2008). From a clinical point of view, a high phenotypic variability is associated with *GRN* mutations, even in the same family (Benussi et al., 2009; Momeni et al., 2010; Pietroboni et al., 2011): specifically, Momeni et al. (2010) studied a Latino family in which SCZ and FTD coexisted, suggesting a molecular link between FTD, SCZ and *GRN* mutations. Another study reported that the morbid risk for SCZ is significantly increased in first-degree relatives of subjects with FTD with respect to first-degree relatives of people with AD; in addition, in three families with a known *GRN* mutation, there was a co-segregation of mutation and SCZ (Schoder et al., 2010). Though the concomitance of FTD and SCZ has been reported in few family pedigrees, the pathogenic mechanism of loss of function *GRN* mutations is consistent with the neurodevelopmental hypothesis of SCZ, as the haploinsufficiency exist throughout development and childhood (Rapoport et al., 2005; Fatemi, 2009). Accordingly, progranulin concentrations were significantly higher in pregnant women compared to post-partum levels (Todoric et al., 2012), confirming its role during embryonic development (Daniel et al., 2003). In this view, psychiatric disorders might represent a long lasting preclinical phase preceding FTD in some *GRN* mutation carriers (Momeni et al., 2010). In line with these data, it’s important to note that two clinically different *GRN* mutated FTLD cases (one with bvFTD and one with progressive non fluent aphasia) showed a premorbid BD status (Cerami et al., 2011).

## Concluding Remarks

In this review, we described how BDNF and progranulin proteins are involved in psychiatric and neurodegenerative diseases. We found that our protagonists share common molecular features in these pathologies. Specifically we noticed that: (i) Circulating BDNF and progranulin protein levels are reduced in pathological conditions: a decrease of BDNF levels was observed in MD and BD as well as in FTLD and related disorders; in parallel, a reduction of progranulin was detected not only in *GRN*-associated FTLD, but also in BD; (ii) for *BDNF* and *GRN* genes, the disease-associated variants cause a reduction in protein levels: the BDNF Val66Met polymorphism, a risk factor for BD and FTLD, results in a impaired secretion of the protein; (iii) in parallel, the whole panel of pathogenic *GRN* mutations as well as polymorphisms cause reduced protein expression or secretion, although by different cellular mechanisms; (iv) both *BDNF* and *GRN* promoters are highly methylated in pathological conditions: a different degree of methylation, and consequently a reduction of gene expression, was observed in *BDNF* and *GRN* promoter regions in BD and FTLD, respectively; and (v) equally BDNF and progranulin are involved in neurodevelopment and neuroplasticity: an insult or a genetic defect might unbalance the expression of these neurotrophins in specific brain regions and in different period of life and thus results in either neurodegenerative or psychiatric diseases.

In this view, we may speculate that, as suggested by Momeni et al. (2010) for subjects with progranulin deficit, psychiatric disorders might represent a preclinical phase preceding FTD.

Given all these similarities, we believe that drug discovery effort aimed at enhancing both BDNF and progranulin levels is of great interest for the prevention and cure of psychiatric and neurological disorders.

## Author Contributions

All authors contributed to the researching of data for the article, and to the review/editing of the manuscript before submission. RZ and MC equally contributed to writing the first draft of the manuscript.

## Conflict of Interest Statement

The authors declare that the research was conducted in the absence of any commercial or financial relationships that could be construed as a potential conflict of interest.
